# Supplementary material for: Exact Solutions of Coupled Multispecies Linear Reaction–Diffusion Equations on a Uniformly Growing Domain
Source: PLoS One. 2015 Sep 25;10(9):e0138894. doi: 10.1371/journal.pone.0138894 (PMC4583548; doi:10.1371/journal.pone.0138894)
Supplement: S1 Supporting Information — (PDF) [file pone.0138894.s001.pdf]

Supplementary Material

Matthew J Simpson[1,*], Jesse A Sharp[1], Liam C Morrow[1], Ruth E Baker[2],

1 School of Mathematical Sciences, Queensland University of Technology, Brisbane, Australia.
1 Mathematical Institute, University of Oxford, Radcliffe Observatory Quarter, Woodstock Road, Oxford, UK.

***matthew.simpson@qut.edu.au**

Numerical Methods

We will now describe how we obtain numerical solutions to the system of coupled equations given by

$$\frac{\partial C_1}{\partial t} = D\frac{\partial^2 C_1}{\partial x^2} - \frac{\partial (vC_1)}{\partial x} - k_1 C_1, \tag{1}$$
$$\frac{\partial C_2}{\partial t} = D\frac{\partial^2 C_2}{\partial x^2} - \frac{\partial (vC_2)}{\partial x} + 2k_1 C_1 - k_2 C_2, \tag{2}$$
$$\frac{\partial C_3}{\partial t} = D\frac{\partial^2 C_3}{\partial x^2} - \frac{\partial (vC_3)}{\partial x} + 2k_2 C_2 - k_3 C_3, \tag{3}$$
$$\frac{\partial C_4}{\partial t} = D\frac{\partial^2 C_4}{\partial x^2} - \frac{\partial (vC_4)}{\partial x} + 2k_3 C_3 - k_4 C_4, \tag{4}$$

on $0 < x < L(t)$, with zero diffusive flux boundary conditions, at both $x = 0$ and $x = L(t)$, for each species. The first step is to apply the boundary fixing transformation $\xi = x/L(t)$, and recall that $v = \xi \mathrm{d}L(t)/\mathrm{d}t$, which allows us to re–write the system as

$$\frac{\partial C_1}{\partial t} = \frac{D}{L^2(t)}\frac{\partial^2 C_1}{\partial \xi^2} - \sigma(t)C_1 - k_1 C_1, \tag{5}$$
$$\frac{\partial C_2}{\partial t} = \frac{D}{L^2(t)}\frac{\partial^2 C_2}{\partial \xi^2} - \sigma(t)C_2 + 2k_1 C_1 - k_2 C_2, \tag{6}$$
$$\frac{\partial C_3}{\partial t} = \frac{D}{L^2(t)}\frac{\partial^2 C_3}{\partial \xi^2} - \sigma(t)C_3 + 2k_2 C_2 - k_3 C_3, \tag{7}$$
$$\frac{\partial C_4}{\partial t} = \frac{D}{L^2(t)}\frac{\partial^2 C_4}{\partial \xi^2} - \sigma(t)C_4 + 2k_3 C_3 - k_4 C_4, \tag{8}$$

on $0 < \xi < 1$, with $\partial C_i/\partial \xi = 0$ at $\xi = 0$ and $\xi = 1$ for $i = 1, 2, 3$ and 4. We discretize Equations (5)–(8), with a central finite difference approximation, on $0 < \xi < 1$, with uniform mesh spacing $\delta\xi$. The resulting system of coupled ordinary differential equations is integrated through time using a backward Euler approximation with uniform time steps of duration δt. The numerical solutions are integrated through time sequentially, treating Equations (5)–(8) separately. The resulting system of tridiagonal linear equations for each component is solved using the Thomas algorithm. All numerical results in the main manuscript are obtained for choices of $\delta\xi$ and δt that produce grid–independent results. The numerical solutions on the fixed domain, $0 < \xi < 1$, are then re–expressed on the growing domain by applying the inverse boundary fixing transformation, $x = \xi L(t)$.

Combined homogeneous Dirichlet and homogeneous flux boundary conditions

In the main part of the manuscript we demonstrate how to solve the governing equations for homogeneous Neumann (no–flux) boundary conditions. It is also possible to solve problems that involve a combination of homogeneous Dirichlet and zero flux boundary conditions, and we will demonstrate this by considering

$$\frac{\partial a}{\partial t} = D\frac{\partial^2 a}{\partial x^2} - \frac{\partial (va)}{\partial x} - ka, \tag{9}$$

on $0 < x < L(t)$ with $\partial a/\partial x = 0$ at $x = 0$, and $a = 0$ at $x = L(t)$. This means that we have zero diffusive flux at the origin, and a homogeneous Dirichlet condition at the moving boundary $x = L(t)$. We proceed in the usual way by applying the boundary fixing transformation, $\xi = x/L(t)$, and then re–scaling the time variable $T(t) = \int_0^t D/L^2(s)\,\mathrm{d}s$, to give

$$\frac{\partial a}{\partial T} = \frac{\partial^2 a}{\partial \xi^2} + f(T)a, \tag{10}$$

on $0 < \xi < 1$ where $f(T) = -L^2(T)(k + \sigma(T))/D$. The boundary conditions in the transformed coordinates are $\partial a/\partial \xi = 0$ at $\xi = 0$, and $a = 0$ at $\xi = 1$. The solution of Equation (10) with these boundary conditions can be found using separation of variables to give

$$a(\xi, T) = \sum_{n=0}^{\infty} \Psi_n \cos(\lambda_n \xi)\mathrm{e}^{-\lambda_n T(t)}\mathrm{e}^{\int_0^T f(T^*)\mathrm{d}T^*}, \tag{11}$$

where $\lambda_n = (2n-1)\pi/2$. In this form, the Fourier coefficients, Ψ_n, can be chosen so that the solution matches the initial condition for $a(\xi, 0)$. Once we determined the Fourier coefficients we can apply the inverse transforms to give $a(x,t)$, as required.

Nonhomogeneous flux boundary conditions

In the main part of the manuscript we demonstrate how to solve the governing equations for homogeneous Neumann (no–flux) boundary conditions, and we also

explain how this procedure can be adapted to deal with homogeneous and nonhomogeneous Dirichlet boundary conditions. However, the adaption of our technique to deal with nonhomogeneous flux boundary conditions is not standard, and we explain the key steps here. If we are interested in solving a coupled system of reaction–diffusion equations on a growing domain with nonhomogeneous flux boundary conditions, we first apply the Sun and Clement transformation to uncouple the system. This will lead to a systems of uncoupled partial differential equations. Each equation in the system has the form

$$\frac{\partial a}{\partial t} = D\frac{\partial^2 a}{\partial x^2} - \frac{\partial (va)}{\partial x} - ka, \tag{12}$$

on $0 < x < L(t)$. Suppose that the boundary condition at $x = 0$ represents a constant diffusive flux of $a(x,t)$ into the domain, $\partial a/\partial x = -J/D$ at $x = 0$, where $J > 0$ is the flux, and the moving boundary at $x = L(t)$ is closed to diffusive fluxes, giving $\partial a/\partial x = 0$ at $x = L(t)$. To solve Equation (12) for $a(x,t)$, we use a boundary fixing transformation, $\xi = x/L(t)$, which, with $v = \xi \mathrm{d}L(t)/\mathrm{d}t$, gives

$$\frac{\partial a}{\partial t} = \frac{D}{L^2(t)}\frac{\partial^2 a}{\partial \xi^2} - (k + \sigma(t))a, \tag{13}$$

on $0 < \xi < 1$. Re-scaling time, $T(t) = \int_0^t D/L^2(s)\,\mathrm{d}s$, gives

$$\frac{\partial a}{\partial T} = \frac{\partial^2 a}{\partial \xi^2} + f(T)a, \tag{14}$$

where $f(T) = -L^2(T)(k + \sigma(T))/D$. The boundary conditions in the transformed coordinate system are $\partial a/\partial \xi = g(T)$ at $\xi = 0$ and $\partial a/\partial \xi = 0$ at $\xi = 1$, where $g(T) = -JL(T)/D$. This means that the constant flux boundary condition in the original physical coordinate system is transformed into a time–dependent flux boundary condition in the transformed coordinate system.

We assume that the solution of Equation (14) can be written as the sum of the solution of a related homogeneous problem and the solution of a related

nonhomogeneous problem

$$a(\xi,T) = \underbrace{v(\xi,T)}_{\text{homogeneous}} + \underbrace{w(\xi,T)}_{\text{nonhomogeneous}}, \tag{15}$$

where choosing

$$w(\xi,T) = \frac{\xi(\xi-2)g(T)}{2}, \tag{16}$$

satisfies the nonhomogeneous boundary conditions for $a(\xi,T)$. After making these choices, $v(\xi,T)$ must satisfy

$$\frac{\partial v}{\partial T} = \frac{\partial^2 v}{\partial \xi^2} + f(T)v(\xi,T) + h(\xi,T), \tag{17}$$

with homogeneous boundary conditions $\partial v/\partial\xi = 0$ at $\xi = 0$ and $\partial v/\partial\xi = 0$ at $\xi = 1$. Here, $h(\xi,T)$ is known, and given by

$$h(\xi,T) = \frac{\partial^2 w}{\partial \xi^2} - \frac{\partial w}{\partial T} + f(T)w(\xi,T). \tag{18}$$

To proceed we assume that both the known function, $h(\xi,T)$, and the unknown function, $v(\xi,T)$, can be written in terms of their cosine eigenfunction expansions

$$v(\xi,T) = \frac{v_0(T)}{2} + \sum_{n=1}^{\infty} v_n(T)\cos(n\pi\xi), \tag{19}$$

$$h(\xi,T) = \frac{h_0(T)}{2} + \sum_{n=1}^{\infty} h_n(T)\cos(n\pi\xi), \tag{20}$$

where

$$h_0(T) = 2\int_0^1 h(\xi,T)\,\mathrm{d}\xi, \tag{21}$$

$$h_n(T) = 2\int_0^1 h(\xi,T)\cos(n\pi\xi)\,\mathrm{d}\xi, \quad n = 1,2,3,\ldots. \tag{22}$$

To solve for $v_0(T)$ and $v_n(T)$, we substitute Equations (21)–(22) into Equation (17),

which gives an infinite system of ordinary differential equations

$$\frac{\mathrm{d}v_0(T)}{\mathrm{d}T} - f(T)v_0(T) = h_0(T),$$
$$\frac{\mathrm{d}v_n(T)}{\mathrm{d}T} + (\pi^2 n^2 - f(T))v_n(T) = h_n(T), \quad n = 1, 2, 3, \ldots, \tag{23}$$

whose solution can be obtained by using an integrating factor

$$v_0(T) = \mathrm{e}^{\int f(T)\,\mathrm{d}T}\left(\int \mathrm{e}^{\int -f(T)\,\mathrm{d}T} h_0(T)\,\mathrm{d}T + c_0\right), \tag{24}$$
$$v_n(T) = \mathrm{e}^{\int f(T)-(n\pi)^2\,\mathrm{d}T}\left(\int \mathrm{e}^{\int (n\pi)^2 - f(T)\,\mathrm{d}T} h_n(T)\,\mathrm{d}T + c_n\right), \quad n = 1, 2, 3, \ldots, \tag{25}$$

where the constants c_0 and c_n can be chosen to ensure that the solution matches the initial condition at $t = 0$,

$$v_0(0) = 2\int_0^1 (a(\xi, 0) - w(\xi, 0))\,\mathrm{d}\xi, \tag{26}$$
$$v_n(0) = 2\int_0^1 (a(\xi, 0) - w(\xi, 0))\cos(n\pi\xi)\,\mathrm{d}\xi, \quad n = 1, 2, 3, \ldots \tag{27}$$

With this information we can write the solution as

$$a(\xi, T) = \frac{v_0(T)}{2} + \sum_{n=1}^{\infty} v_n(T)\cos(n\pi\xi) + w(\xi, T). \tag{28}$$

This solution in the transformed coordinate system, $a(\xi, T)$, can be rewritten in the original physical coordinate system to give $a(x, t)$. This framework for constructing the exact solution can be applied to a general $L(t)$, and we will now evaluate the solution for an exponentially elongating domain, $L(t) = L(0)\mathrm{e}^{\alpha t}$ with $\alpha > 0$. We will also compare the exact solution with approximate numerical solutions. To generate the numerical solution we discretize Equation (13) using a central finite difference approximation on a uniformly discretized domain, $0 < \xi < 1$, with uniform mesh spacing $\delta\xi$. The boundary conditions at $\xi = 0$ and $\xi = 1$ are implemented by discretizing the flux conditions at the boundary nodes using a forward and backward difference approximation, respectively. This leads to a system of coupled ordinary differential equations which we integrate through time using a backward Euler approximation with uniform time steps of duration δt. At each time step the system of

tridiagonal equations is solved using the Thomas algorithm. For all numerical solutions we always choose $\delta\xi$ and δt to be sufficiently small so that the numerical solutions are grid–independent.

For exponential growth we have $\sigma(t) = \alpha$ and

$$T(t) = \frac{D(1-\mathrm{e}^{-2\alpha t})}{2\alpha L^2(0)}, \tag{29}$$

$$t(T) = -\frac{1}{2\alpha}\log_{\mathrm{e}}\left(1 - \frac{2\alpha L^2(0)T}{D}\right). \tag{30}$$

To simplify the algebra we define $\kappa = 2\alpha L^2(0)/D$, $\nu = -L^2(0)(k+\alpha)/D$ and $\Omega = JL(0)/D$. For this problem we have $f(T) = \nu/(1-\kappa T)$ and $g(T) = -\Omega/\sqrt{1-\kappa T}$ and the solution of the corresponding nonhomogeneous problem is

$$w(\xi, T) = \frac{(\xi-2)\xi\Omega}{2\sqrt{1-\kappa T}}. \tag{31}$$

It is important to note that as $t \to \infty$ we have $T(t) \to (1/\kappa)^-$, so that $1 - \kappa T > 0$ for all $t > 0$. This means that $f(T)$, $g(T)$ and $w(\xi, T)$ are all well–defined for all $t > 0$. With the simplest possible initial condition, $a(x, 0) = 0$, we obtain

$$\begin{aligned} v_0(T) &= \frac{2\Omega}{3(\kappa+2\nu)}(1-\kappa T)^{-(2\nu-\kappa)/(2\kappa)} \times \\ &\left[(\kappa+2\nu+6\kappa T-6)(1-\kappa T)^{\nu/\kappa} + 6\sqrt{1-\kappa T}\right], \end{aligned} \tag{32}$$

$$\begin{aligned} v_n(T) &= \frac{\mathrm{e}^{-\pi^2 n^2 T}(T\kappa-1)^{-\nu/\kappa}\,\Omega}{n^2\pi^2\kappa\sqrt{1-\kappa T}}\left[\mathrm{e}^{\pi^2 n^2/\kappa}(\kappa-2\nu) - 2(-1)^{\nu/\kappa}\kappa\sqrt{1-T\kappa}\right] \times \\ &\left[(-1)^{\nu/\kappa}\sqrt{1-T\kappa}\,E_{\frac{3}{2}-\frac{\nu}{\kappa}}\left(\frac{\pi^2 n^2}{\kappa}\right) - (T\kappa-1)^{\nu/\kappa}\,E_{\frac{3}{2}-\frac{\nu}{\kappa}}\left(\frac{\pi^2 n^2 \pi^2(1-T\kappa)}{\kappa}\right)\right], \\ &\quad n = 1, 2, 3, \ldots, \end{aligned} \tag{33}$$

where $E_s(z) = \int_1^\infty \mathrm{e}^{-zt}/t^s \,\mathrm{d}t$ is the exponential integral [1]. With this information the solution can be written as

$$a(\xi, T) = \frac{v_0(T)}{2} + \sum_{n=1}^{\infty} v_n(T)\cos(n\pi\xi) + \frac{(\xi-2)\xi JL(0)}{2D\sqrt{1-(2\alpha L^2(0)T)/D}}, \tag{34}$$

which can be rewritten in the original coordinate system, $a(x, t)$, by recalling that $T(t) = (1-\mathrm{e}^{2\alpha t}D)/(2\alpha L^2(0))$ and $\xi = x/(L(0)\mathrm{e}^{\alpha t})$.

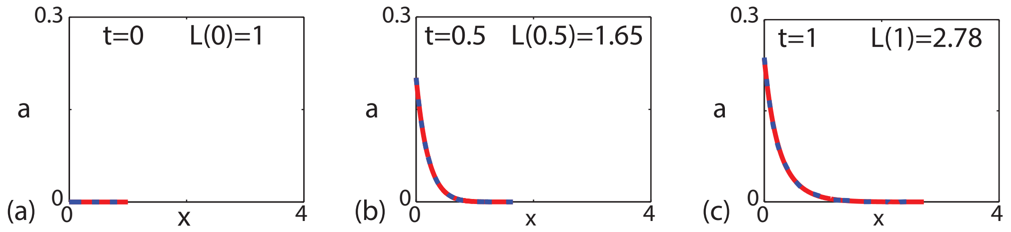

Figure 1. (a)–(c) Exact (red solid) and numerical (blue dashed) solutions of Equation (9) an exponentially growing domain with $L(0) = 1$, $\alpha = 1$, $D = 0.1$, $J = 0.1$ and $k = 1$. Exact solutions are obtained by truncating the infinite series after 10 terms. Numerical solutions correspond to $\delta\xi = \delta t = 2 \times 10^{-10}$. All exact calculations, including the evaluation of the Exponential integral, are evaluated using Mathematica [2].

We now compare numerical and exact solutions of Equation (12), on an exponentially growing domain in Figs S1(a), (b) and (c) confirming that the exact and numerical solutions are visually indistinguishable.

Although the eigenfunction expansion technique described here produces an exact solution for an exponentially growing domain with a simple initial condition, $a(x, 0) = 0$, we have found that applying the technique to other choices of $L(t)$ and $a(x, 0)$ can lead to intractable integrals in the definition of $v_0(T)$ and $v_n(T)$.

Approximating nonlinear models by linearised models

A common theme in the Introduction and Discussion sections of the main document is that one of the key limitations of our work is that the exact solution strategy is valid only for linear partial differential equations whereas many reaction–diffusion models used to represent collective cell spreading processes are nonlinear. We will now demonstrate the ability of a linear reaction–diffusion equation to approximate the solution of a related nonlinear reaction–diffusion equation on a growing domain. To demonstrate this we will consider a single, uncoupled, reaction–diffusion equation on a growing domain,

$$\frac{\partial C}{\partial x} = D\frac{\partial^2 C}{\partial x^2} - \frac{\partial (vC)}{\partial x} + kS(C), \tag{35}$$

where D is the diffusivity, v is the advective velocity associated with domain growth, k is the proliferation rate and $S(C)$ is a generalised source term. Setting $S(C) = C$ means that we are considering a linear source term whereas setting $S(C) = C(1 - C)$ is a logistic-type nonlinear source term. This kind of nonlinear source term is often used to mimic crowding effects since we have $S(1) = 0$ which means that the proliferation rate becomes zero when the density increases to the carrying capacity density, which here we have set to be unity. To demonstrate the differences between setting $S(C) = C$ and $S(C) = C(1 - C)$ we will obtain numerical solutions of Equation (35). When we set $S(C) = C(1 - C)$, numerical solutions of the nonlinear equation are obtained by using an iterative fixed-point method [3].

Results in Figs S2(a), (b), (c) and (d) show the solution of Equation (35) with a linear proliferation term, $S(C) = C$. In comparison, results in Figs S2(e), (f), (g) and (h) show the solution of Equation (35) with a nonlinear proliferation term, $S(C) = C(1 - C)$. All parameter values, boundary conditions and initial conditions used to solve Equation (35) to generate the linear solutions in Figs S2(a), (b), (c) and (d) are exactly the same as those used to solve Equation (35) to generate the nonlinear solutions in Figs S2(e), (f), (g) and (h). Therefore, the differences between the solutions in Figs S2(a), (b), (c) and (d) and Figs S2(e), (f), (g) and (h) reflect the choice of a linear or nonlinear source term. Comparing results in Figs S2(a), (b), (c)

and (d) with the results in Figs S2(e), (f), (g) and (h) it is clear that, particularly by $t = 10$, the solutions of the linear and nonlinear equations are visually distinct in those regions of the domain where $C(x, t)$ is sufficiently large. However, if we focus on the low density region of the solutions, as shown in Figs S2(i), (j), (k) and (l), we see that the solution of the linear model provides an excellent approximation of the solution to the nonlinear model. Therefore, while we acknowledge that the linear model provides a poor approximation of the solution of the nonlinear model in the high–density region of the domain, the linear model provides an excellent approximation of the nonlinear model in the low–density region of the domain. Therefore, if the aim of using a mathematical model is to study the position of the leading edge of the spreading cell population, we conclude that a linear model can provide useful information. Although we have demonstrated how a linear model can be used to approximate a nonlinear model using a single, uncoupled, linear reaction–diffusion equation, similar results are valid for systems of coupled, linear reaction–diffusion equations on growing domains.

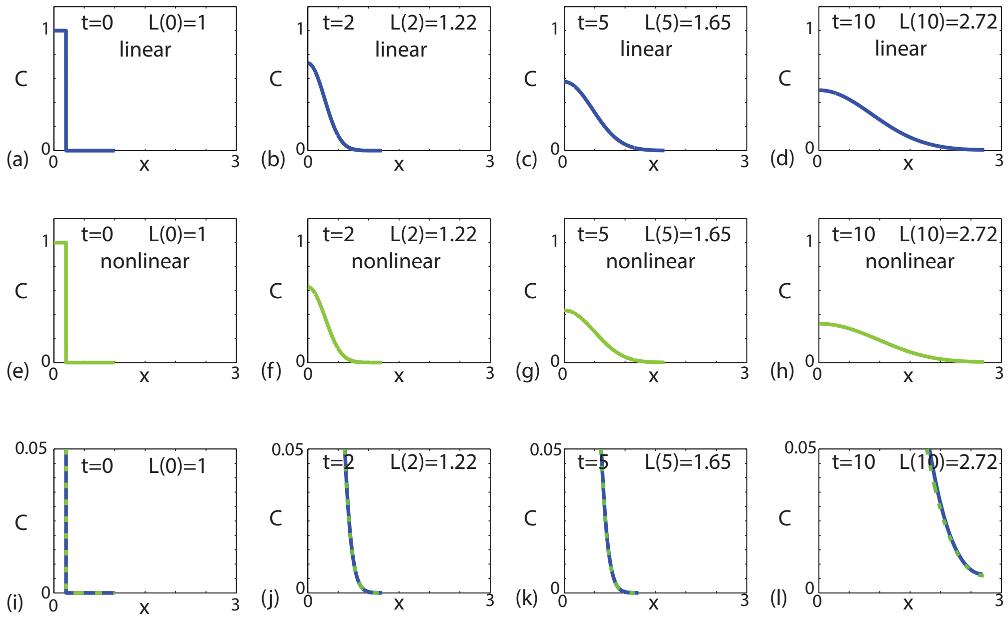

Figure 2. (a)–(d) Solutions of Equation (35) on a growing domain with a linear source term, $S(C) = C$ (solid blue). (e)–(h) Solutions of Equation (35) on a growing domain with a nonlinear source term, $S(C) = C(1 - C)$ (solid green). Profiles in (i)–(l) compare the linear solutions from (a)–(d) (solid blue) with the nonlinear solutions in (e)–(h) (dashed green) at the leading edge of the population where $C(x,t) < 0.05$. Solutions are shown at $t = 0, 2, 5$ and 10, as indicated. All cases correspond to an exponentially growing domain with $L(0) = 1$, $\alpha = 0.1$, $D = 0.01$, $k = 0.1$ and $\gamma = 0.2$. Numerical solutions correspond to $\delta\xi = \delta t = 0.001$.

References

1. Abramowitz M, Stegun IA (1972) Handbook of mathematical functions with formulas, graphs, and mathematical tables. New York. Dover.

2. Wolfram Research Inc (2014) Mathematica. Wolfram Research Inc. Champaign, Illinois.

3. Morton KW, Mayers DF (2005) Numerical solution of partial differential equations. Cambridge University Press. Cambridge.
